# Supplementary material for: Canalized light creates directional and switchable surface structures in vanadium dioxide
Source: Nat Commun. 2025 Apr 28;16:3960. doi: 10.1038/s41467-025-58929-6 (PMC12037821; doi:10.1038/s41467-025-58929-6)
Supplement: Supplementary file 1 — Supplementary Information [file 41467_2025_58929_MOESM1_ESM.pdf]

Supplementary Information: Canalized Light Creates Directional and Switchable Surface Structures in Vanadium Dioxide

Daniel Kazenwadel†, Noel Neathery†, Peter Baum*

Universität Konstanz, Fachbereich Physik, 78464 Konstanz, Germany

†These authors contributed equally to this work.

**peter.baum@uni-konstanz.de*

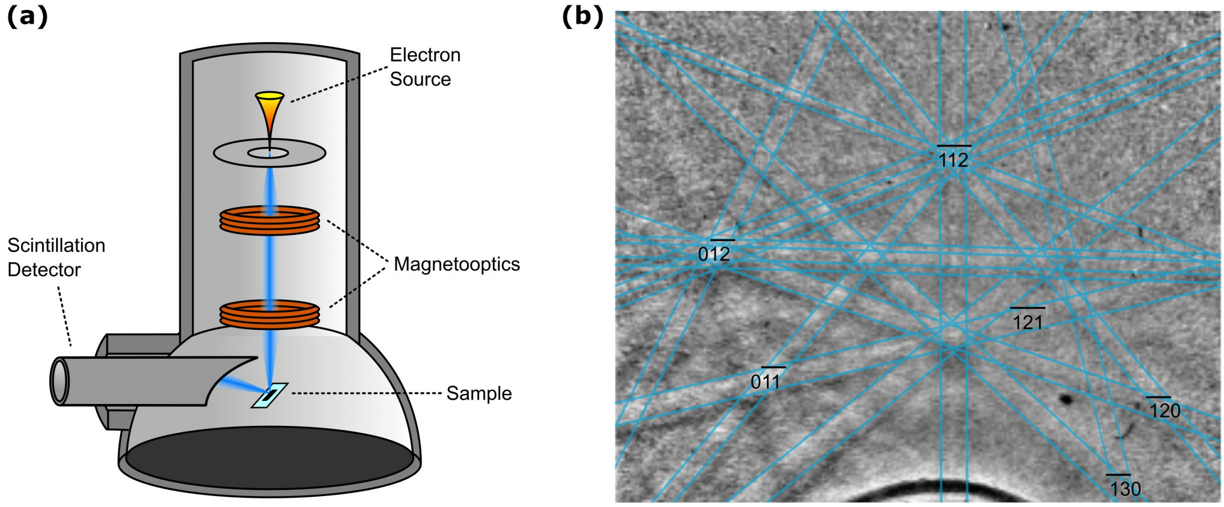

Supplementary Figure 1. Electron backscatter diffraction on 800 nm grooves. (a) The electron beam has an energy of E_{HV} = 20 keV and hits the sample at an angle of 70° close to grazing incidence. The grooves are aligned orthogonal to the plane of incidence of the electrons, therefore the beam probes only the patterned ridges and not the substrate. The backscattered electrons are then detected by a scintillation detector. **(b)** Backscattered electron diffraction data with indexing of the most visible features. All visible features can be indexed properly but not all labels are plotted in the figure for clarity. We see clear Kikuchi lines that confirm that the written grating is indeed mostly single-crystalline. The patterned and uneven surface causes some blurring in comparison to the unthreatened surface (*65*).

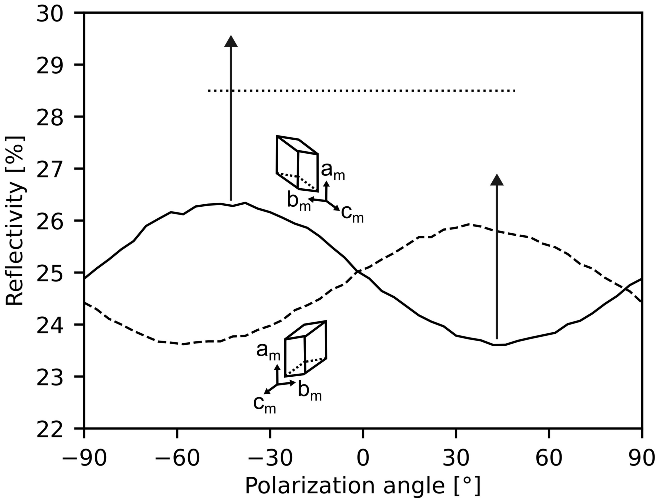

Supplementary Figure 2. Optical anisotropy of our VO_2 single crystals. Polarization-dependent reflectivity of a twin tilted to the left (solid line) and a twin tilted to the right (dashed line). Zero degree denotes the angle parallel to c_r. In the low temperature phase, the maximum reflectivity is always aligned in the direction of the c_m axis. Arrows indicate that general effects of photodoping. Metallicity (symbolic dotted line) is reached earlier and more efficiently along $\mathbf{c}_m$. Measurements of transient reflectivity in nanostructures (*27*) are compatible with these result, because data was measured along the high temperature $\mathbf{c}_r$ axis, where the 3D-rotated unit cell of VO_2 (*54*) results in a vanishingly small projection of the anisotropy.
